# Distance-dependent effects on CRISPR/Cas9-mediated genome editing in *Schizosaccharomyces pombe* compromise efficiency and create unsought alleles

**DOI:** 10.17912/micropub.biology.001248

**Published:** 2024-07-25

**Authors:** Reine U Protacio, Emory G Malone, Wayne P Wahls

**Affiliations:** 1 Biochemistry and Molecular Biology, University of Arkansas for Medical Sciences, Little Rock, Arkansas, United States

## Abstract

Discrete DNA sites position meiotic recombination at hotspots. We sought to create four different, 15 bp long, candidate regulatory DNA sites within the *ura4* reporter gene. Each effort employed a fission yeast-optimized CRISPR system (SpEDIT), optimal guide RNA, and one of four homologous recombination templates with 10 to 15 bp substitutions. Remarkably, every Ura ^-^ transformant analyzed had template-directed, PAM-disabling bp substitutions near (5-6 bp away from) the DSB but no DNA site-generating substitutions at distance (42-56 bp). An unsought novel allele, ura4-P127* , has two substitutions (C379T, C380A) that create a stop codon, rendering strains unable to grow without uracil.

**Figure 1. Approach for genome editing, resulting mutations, and phenotypes of mutants.**
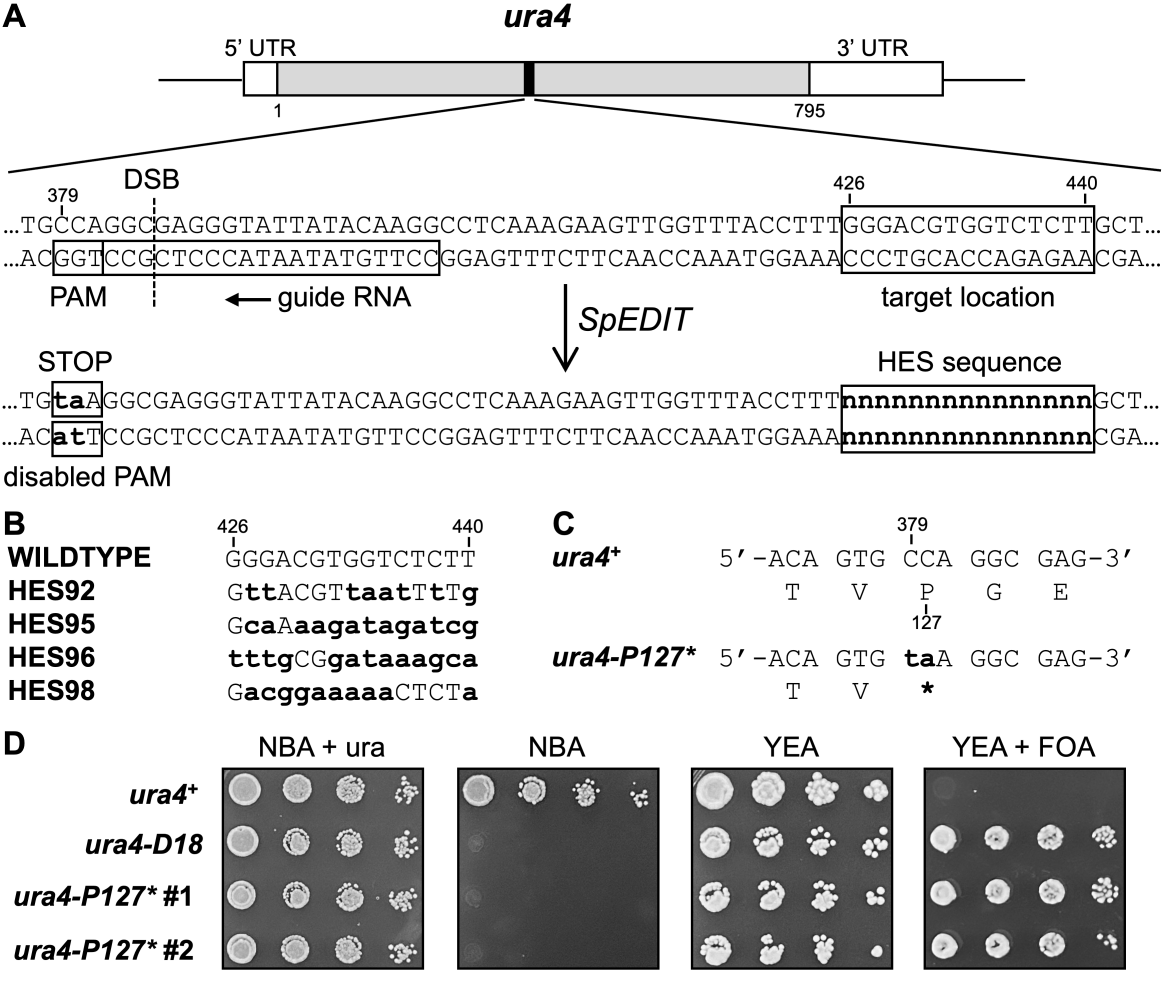
**A.** Scale diagram of the *ura4* gene and relevant DNA sequences; coordinates are relative to the first nucleotide of the start codon (+1). Positions of the guide RNA, PAM, Cas9-induced dsDNA break (DSB), and target location for newly created DNA sites are indicated. Successful editing (SpEDIT) should introduce bp substitutions (bold lowercase) to simultaneously inactivate PAM and introduce a stop codon (left) and to create a HES DNA sequence (right). **B.** Sequences of HES DNA elements and bp substitutions needed to create those DNA sequences. **C.** Sequence of ura4-P127* allele shows bp substitutions that create a stop codon for translation (asterisk). **D.** Phenotypes of ura4-P127* cells. Serial dilutions (1:10 per step) of cells were plated on NBA minimal media that contains or lacks uracil (ura) and on YEA rich media that contains or lacks FOA. Two independent transformants harboring ura4-P127* are shown; cells with a wildtype allele ( *ura4 ^+^* ) and a deletion of the gene ( *ura4-D18* ) provide controls.

## Description

The DNA site-dependent control of meiotic recombination hotspots, which collectively regulate the frequency distribution of meiotic recombination across the genome, was discovered first in fission yeast using the *ade6* gene to measure recombination rates [see (Storey *et al.* 2018; Mukiza *et al.* 2019; Protacio *et al.* 2022) and refs therein]. Hotspot-activating DNA sites first discovered and thoroughly characterized at *ade6* were subsequently shown to help position recombination elsewhere in the genome (Wahls and Davidson 2010) . Moreover, an insightful genetic screen of randomized DNA sequences (15 bp or 30 bp in length) revealed hundreds of additional, candidate regulatory DNA elements, based on a qualitative assay for recombination between *ade6* alleles in a plasmid and the chromosome (Steiner *et al.* 2009). We sought to test directly whether four of those DNA sequence elements (called “HES” sequences) promote meiotic recombination between homologous chromosomes. We chose to do so at the *ura4* locus because this gene supports positive and negative selection (Grimm *et al.* 1988) and has been used successfully to confirm the functionality of other hotspot-activating DNA sites (Fox *et al.* 1997). To avoid changing the overall structure or spacing of elements in the genome, we chose to use bp substitutions to create each new allele ( **Figure 1A -1B** ). We selected a fission yeast-optimized CRISPR-Cas9 system called “SpEDIT” for genome editing based on its ease of use and its reportedly high efficiency and precision for editing the *ura4* gene (100%; n = 150) (Torres-Garcia *et al.* 2020).

Our objective was to generate specific, 15 bp-long DNA sequences ( **Figure 1B** ) near the middle of the *ura4* ORF ( **Figure 1A** ). In the SpEDIT system, the guide RNA (gRNA) and a fission yeast codon-optimized Cas9 protein are co-expressed from the same plasmid (Torres-Garcia *et al.* 2020). We used the CRISPR4P program (Rodriguez-Lopez *et al.* 2016) to design the most unique (optimal) gRNA for *ura4* when compared to the genome ( **Figure 1A** ). The same gRNA-Cas9-expressing plasmid was used for each of the allele replacements. For each of the four different allele replacements, we constructed a dsDNA, homologous recombination (HR) template for repair. The 200 bp-long HR templates contained between 10 and 15 centrally located bp substitutions, relative to wild-type *ura4* . Two of these bp substitutions were designed to destroy the sequence of the protospacer adjacent motif (PAM) during the first round of editing ( **Figure 1A** ), thus preventing the genomic target locus from undergoing multiple cycles of Cas9-catalyzed endonucleolytic cleavage. Those two bp changes were also designed to introduce a stop codon, thereby rendering the cells auxotrophic for uracil (Ura ^-^ ). Including this null mutation served two purposes: first, it facilitated the screening of transformants for those with template-directed genome editing; second, it provided a genetic marker for subsequent assays of meiotic recombination. The four HR template DNAs (one per allele) also contained bp substitutions designed to generate a 15 bp long HES DNA sequence ( **Figure 1B** ) within the *ura4* ORF ( **Figure 1A** ). Thus, after Cas9 catalyzes the gRNA-directed formation of a recombination-initiating DSB within *ura4* in the genome, repair of that break from the HR template molecule should simultaneously inactivate the PAM site (via editing 5-6 bp away from the DSB) and introduce the HES DNA sequence element (via editing 42-56 bp away from the DSB).

For each HR template DNA molecule, we co-transformed wild-type ( *ura4 ^+^* ) cells with that HR template and the gRNA-Cas9-expressing plasmid, selected for G418-resistant colonies, and patched those colonies onto non-selective media to allow plasmid loss. We then replica plated the candidates onto minimal media that contained or lacked uracil to score for those that had become uracil auxotrophs (Ura ^-^ ). Of the 300 clones analyzed from the four separate transformations, 69% were Ura ^-^ . We then sequenced the *ura4* locus from some of the Ura ^-^ clones and found that all of them (100%, n = 12) had the desired, template-directed changes at the PAM site. We conclude that our “real-world” application of SpEDIT is somewhat less efficient (only 69% of transformant colonies were rendered Ura ^-^ ) but is as precise (100% of mutants had the designed bp changes) as reported previously (Torres-Garcia *et al.* 2020)—at least for introducing modifications very close to (5-6 bp away from) the recombination-initiating DSB.

The target location in wild-type *ura4* ( **Figure 1A** ) contains a *Bsa* I restriction endonuclease recognition sequence, and that restriction site would be removed if any of the HES DNA sequences is generated at that location. Therefore, we were able to use PCR and *Bsa* I digestion to screen for successful bp substitutions within the target location. Remarkably, all 48 of the Ura ^-^ clones tested retained their *Bsa* I cut site, demonstrating unambiguously that none (0%, n = 48) of those clones had successfully incorporated the template-directed bp changes. This was confirmed by sequencing the *ura4* locus of 12 Ura ^-^ clones: none of them had any template-directed changes at the target site—even though all of them had template-directed changes at PAM.

Although we were unsuccessful at introducing HES DNA sequences into the *ura4* locus, our results provide striking, unexpected insight into the process of CRISPR-Cas9-mediated precise genome editing in fission yeast: SpEDIT can efficiently introduce edits close to the DSB but those proven template-dependent repair events seldom extend over a distance of about 50 bp from their sites of initiation. Thus, real-world applications (such as ours) will need to consider the distance-dependent effects. It would be interesting to see what distance from the cut site is the practical limit; how the length of the HR template affects outcomes; and whether relaxing the “uniqueness” of gRNAs in order to position the DSB closer to the desired edits would be beneficial or detrimental. The choice of CRISPR system (there are several for fission yeast) might have an impact, as might the highly variable efficiencies of editing at different loci (Fernandez and Berro 2024) . Off-target effects of CRISPR are always a concern, as are the on-target insertions of ectopic DNA that happen in fission yeast (Longmuir *et al.* 2019). Given these issues, prudent researchers might elect to use classical allele replacement methods that can efficiently introduce the desired changes over thousands of bp in the genome, without off-target effects (Gao *et al.* 2014; Storey *et al.* 2019).

Lastly, while we did not intend to create the ura4-P127* allele, this centrally located mutation in *ura4* might be of utility to other scientists. We therefore tested the ability of ura4-P127* mutants to grow on media that lacks uracil (which requires a wild-type *ura4* gene) and on media that contains 5-fluroroorotic acid (which selects for cells that lack a functional *ura4* gene) (Grimm *et al.* 1988; Protacio *et al.* 2024). As expected from the fact that ura4-P127* cells do not express full-length Ura4 protein ( **Figure 1C** ), the phenotypes of the mutants were identical to those of the null mutant control ( **Figure 1D** ).

## Methods

Standard methods were used to culture fission yeast using yeast extract agar (YEA: 5 g/L VWR Life Sciences yeast extract, 3% Sigma glucose, 1.8 % Fisher Bioreagents agar) or liquid as the rich medium and nitrogen base agar (NBA: 1.7 g/L Sunrise Science Products yeast nitrogen base without amino acids, 5 g/L Amresco ammonium sulfate, 1% Sigma glucose, 1.8 % Fisher Bioreagents agar) or liquid for minimal medium (Gao et al. 2014; Storey et al. 2019) . Media were supplemented as required with uracil (100 µg/ml) or 5-F0A (1 mg/ml) (Grimm et al. 1988) . Spot tests were performed by preparing 10-fold serial dilutions (5 x 10 ^5^ to 5 x 10 ^2^ cells/ml) of strains and then using a hanging drop pinner to transfer 10 µl of each dilution to plates. All cultures were grown at 32°C. The *SpEDIT* system was used to incorporate changes into the *ura4* ^+^ gene using published protocols (Torres-Garcia et al. 2020) . Briefly, the strain WSP3776 was transformed with the plasmid pLSB- *kanMX6* loaded with the chosen sgRNA together with the HR template bearing the desired mutations. Each HR template was synthesized by annealing two 100 bp-long oligonucleotides (HES-F and HESnn-R) and filling in the gaps with a high-fidelity polymerase. Following gel-purification of the correctly sized product, two short primers (ura4-F2 and ura4-R2) were used to amplify the HR fragment for use in transformations. For diagnostic PCR and sequence analysis, genomic DNA samples were prepared using the “smash and grab” method with cells from 5 ml of an overnight culture. PCR and DNA sequencing were conducted using the oligonucleotide primers ura4FOR and ura4REV.

## Reagents

**Table:** 

Oligonucleotides:	
**Name**	**Sequence**
ura4 sgRNA-F	5'-CTAGAGGTCTCGGACTCCTTGTATAATACCCTCGCCGTTTCGAGACCCTTCC-3'
ura4 sgRNA-R	5'-GGAAGGGTCTCGAAACGGCGAGGGTATTATACAAGGAGTCCGAGACCTCTAG-3'
HES-F	5'-ATTCGCAGACATTGGAAATACCGTCAAGCTACAATATGCATCTGGTGTGTACAAAATTGCTTCTTGGG CTCATATCACAAATTGCCATACAGTGTAAGGC-3'
HES92-R	5'-TTGGAAGACATTTCAGCCAAAAGCCAAAATTAACGTAACAAAGGTAAACCAACTTCTTTGAGGCCTTG TATAATACCCTCGCCTTACACTGTATGGCAAT-3'
HES95-R	5'-TTGGAAGACATTTCAGCCAAAAGCCGATCTATCTTTTGCAAAGGTAAACCAACTTCTTTGAGGCCTTG TATAATACCCTCGCCTTACACTGTATGGCAAT-3'
HES96-R	5'-TTGGAAGACATTTCAGCCAAAAGCTGCTTTATCCGCAAAAAAGGTAAACCAACTTCTTTGAGGCCTTG TATAATACCCTCGCCTTACACTGTATGGCAAT-3'
HES98-R	5'-TTGGAAGACATTTCAGCCAAAAGCTAGAGTTTTTCCGTCAAAGGTAAACCAACTTCTTTGAGGCCTTG TATAATACCCTCGCCTTACACTGTATGGCAAT-3'
ura4-F2	5'-GGATCGCAAATTCGCAGAC-3'
ura4-R2	5'-CCAAAGAGCCTTTGGAAGAC-3'
ura4 FOR	5'-CCATCCCAGTTTAACTATGCTTCGTC-3'
ura4 REV	5'-CGCCTAGGAAAACAAACGCAAACAA-3'

**Table:** 

Fission yeast strains:		
**Name**	**Genotype**	**Source**
WSP 3776	* h ^-^ wildtype *	Wahls Lab
WSP 3803	* h ^-^ ura4-D18 *	Gould Strain KGY600
WSP 8490	h ^-^ ura4-P127*	This study
WSP 8491	h ^-^ ura4-P127*	This study
